# Challenges and lessons learned in mental health research among refugees: a community-based study in Turkey

**DOI:** 10.1186/s12889-021-11571-5

**Published:** 2021-08-11

**Authors:** Ozge Karadag, Cengiz Kilic, Edip Kaya, Sarp Uner

**Affiliations:** 1 Center for Sustainable Development, Earth Institute, Columbia University, NY New York, USA; 2 STAR (Stress Assessment and Research Center), Hacettepe University, Ankara, Turkey; 3 Department of Psychiatry, Faculty of Medicine, Hacettepe University, Ankara, Turkey; 4 Department of Public Health, Institute of Health Sciences, Hacettepe University, Ankara, Turkey; 5 Department of Disabled Care and Rehabilitation, Vocational School of Health Services, Agri Ibrahim Cecen University, Agri, Turkey; 6 Institute of Public Health, Hacettepe University, Ankara, Turkey

**Keywords:** Mental health, Trauma, Forced displacement, Refugee, Community-based research, Urban setting

## Abstract

**Background:**

Turkey hosts nearly four million refugees and 99% live in urban areas. Research in urban settings pose different challenges and opportunities than research in refugee camps. In this article, we aimed to share the challenges and experiences we encountered in a mixed-methods study to assess mental health problems and barriers to accessing mental health care among refugees in urban areas of Turkey.

**Discussion:**

In our case, the main challenges in conducting research with refugees were collecting data from a highly traumatized population, difficulties with contacting undocumented asylum seekers including trust issues and the fear of deportation, the risk of secondary traumatization among data collectors, and the bureaucracy during study approval processes. Targeting a representative sample was not feasible, because of the lack of publicly available demographic data on a district level, presence of undocumented asylum seekers and high mobility among the refugees. Although respondents with significant psychological symptoms were routinely referred to available mental health services, we were able to do less for unregistered refugees with problems in accessing health care. Language/alphabet differences and differing dialects of Arabic posed another challenge in both translation and administration of the scales. Based on cultural characteristics, a gender-balanced team was used and the interviewers were gender-matched whenever needed. Also, the research team had to work after work hours and during weekends to be able to interview male refugees, since most refugee men were at work during working hours and most days of the week.

**Conclusions:**

The research team’s experience showed that refugee population characteristics including level of trauma, language, culture, gender, legal status, and urban setting characteristics including places of living, mobility, availability of publicly available demographic data, and outreach-related barriers lead to different challenges and ethical responsibilities of researchers and affect the research costs in terms of time, human resources and finance. Even in a host country with geographical, religious and cultural proximity to the refugees, profound challenges exist in conducting mental health research in urban settings. Learning from previous experience and collaborating with local researchers and institutions are vital for better public health research and practice outcomes.

## Background

### Humanitarian context

According to the UN Refugee Agency (UNHCR), more than 6 million refugees fled Syria because of the ongoing war [1]. Refugees from conflict zones face numerous problems in host countries due to loss of social support systems, poor living conditions, and barriers in accessing services, in addition to the effects of past traumas in their home countries [2–4]. Studies show that refugees are highly traumatized and mental health problems are common, with post-traumatic stress disorder (PTSD) and depression being especially prevalent [3–7]. According to literature, living in urban settings pose different challenges and opportunities for refugees when compared to living in camps [8].

Turkey hosts nearly four million refugees and 99% live in urban areas [1, 9]. Literature review showed that most of the previous mental health research in the country reflected camp settings with comparatively better organization of services including health care [2, 7, 10], and studies about mental health status and/or access to mental health services for those living in urban areas were scarce. Those few studies that were conducted in urban areas were in cities close to the Turkey-Syria border, which shared some common characteristics with conflict zones when compared to the cities in other parts of the country (e.g. sounds of aircrafts and bombings across the border, occasional firing of mortar bombs, etc., which might have reminded previous traumas) [11, 12]. Therefore, more scientific evidence was needed to better inform future policies on mental health and mental health care for urban refugee populations.

Research in urban settings pose different challenges and opportunities than research in refugee camps. In this research in practice article, we aimed to share the challenges and experiences we encountered in a mixed-methods study that we conducted to assess mental health problems and barriers to accessing mental health care among refugees in urban areas of Turkey.

## Research study

### Method and key findings

The study described in this research in practice article, used a mixed-methods approach and took place in Ankara, the capital of Turkey, in 2016. Quantitative data were collected by a face-to-face survey among Syrian refugees, by a team of specially trained, bilingual (Turkish-Arabic) university graduate interviewers. The assessment batch included Harvard Trauma Questionnaire (HTQ), Beck Depression Inventory (BDE), in addition to items on socio-demographics, perceived mental health needs, and access to mental health services. The HTQ was developed as a cross-cultural, clinician-administered instrument to assess trauma and torture related to mass violence and their psychological impacts. The Arabic version of the scale was validated by Shoeb et al. [13, 14]. The Beck Depression Inventory (BDI) (Beck et al., 1961) is a widely used self-report questionnaire that measures depressive symptomatology for the last week [15]. Validity and reliability in Arabic were established by West (1985) and Abdel-Khalek (1998) [16, 17].

During quantitative data collection, a total of 420 adult refugees from 229 households located in formal settlements in Ankara were surveyed using snowball sampling method and visiting households. For all participants, Turkey was the first settlement after Syria. Some of them temporarily stayed in camps or in other cities in Turkey before moving to Ankara. Qualitative data were collected via in-depth interviews with 10 health care providers and 10 health policy makers, who had direct experience working for refugees or refugee health policies in Turkey. The interviewers were trained by senior researchers before data collection. The senior researchers accompanied the interviewers and supported data collection in the field.

The study was supported by Hacettepe University Scientific Research Projects Coordination Unit. Ethical approval was obtained from the Ethics Commission of Hacettepe University and institutional approvals from the Ministry of Interior, Department General of Migration Management and the Ministry of Health to work with Syrian refugees and health professionals, respectively.

In summary, the study showed that the community-based study group was highly traumatized: 88.8% reported having witnessed war or armed conflict, 44.0% reported having lost a family member; and 31.1% reported having witnessed a killing. The prevalence of probable PTSD (post-traumatic stress disorder) and depression were found to be 36.5 and 47.7%, respectively. Among the refugees who needed mental health care, only 9.7% were found to have used mental health services. The most common barriers to mental health care were language problems and lack of knowledge about existing services. From the service providers’ and policy makers’ point of view, the reasons for low utilization of mental health services were refugees’ higher prioritization of daily life challenges and physical health problems and their low level of awareness on available services [18–21].

### Scientific importance of research in forced migration settings

Forced displacement is currently one of the most important global challenges. UNHCR data show that over 70 million people are forced to leave their homes due to conflict, war, or oppression. Out of all forced displacements, the number of refugees has risen to a record high of nearly 26 million in the world [22], where the negative effects of wars on civilian populations are already well-known [2, 3].

One of the most important public health priorities for international organizations, academia and governments is to detect those who are most vulnerable to the effects of war and resulting forced displacement. It is also of utmost urgency to assess and follow-up refugees’ mental health problems and barriers in accessing health care in host countries, so that evidence-based policymaking and needs-based service provision can be possible.

The results of this study were expected to inform the public health authorities, humanitarian and non-governmental organizations in Turkey on how to allocate resources in terms of best management for the mental health problems of refugees in urban settings. Our most interesting finding was that mental disorder rates in urban settings were similar to those found in studies conducted in camps. We would expect that refugees who settled in a large city such as Ankara, rich with opportunities and away from the reminders of the war, should have showed lower rates of psychopathology. In addition, our study was conducted much later than the studies conducted in camps; a decline in mental disorder rates would have been expected. This finding points to the need to examine social determinants of mental health and other factors that the refugees go through in host communities in urban settings, which either create new mental problems or prevent the resolution of the existing war-related ones. Through assessments such as the ones used in the present study, it was also possible to learn about factors that affect resilience to the effects of war. Comparison of the effects of direct exposure to war trauma versus the effects of negative life events imposed by the difficulties during forced displacement and during the adaptation process in the host country may have broader implications [23–27].

### Challenges faced by researchers

#### Working with a highly traumatized population

The high prevalence of past and present traumas required better communication skills and resolution of trust issues between the respondents and the interviewers. The interviews lasted longer for those with higher levels of trauma and sometimes caused interviewers to be emotionally affected. At those times, the interviews were supported by senior researchers to decrease the risk of secondary traumatization.

#### Legal status, lack of data, and high mobility of refugees

Obtaining an appropriate sample to study was problematic and targeting a representative sample was not feasible, because of lack of neighborhood-specific data, the presence of undocumented asylum seekers, and high mobility among the registered refugee community. According to official estimates at the time of the study, there were 88.000 Syrian refugees living in Ankara. Since there has been high mobility among the refugees in Turkey, it was not possible to exactly locate the neighborhoods they lived in. Two neighborhoods, known to be densely populated by Syrian refugees, were targeted for the study. At the time of the study, demographic data were not available at a district level and the research team didn’t know how many Syrian refugees lived in those neighborhoods, because of the lack of publicly available data and the presence of unregistered asylum seekers.

#### Safety issues and willingness to participate

Presence of unregistered asylum seekers also led to problems with willingness to participate, since undocumented or unregistered migrants tend to avoid non-essential contact with professionals in host countries because of the fear of deportation. Although non-response rate was very low, some refugees and asylum seekers did not want to participate in the study because of safety concerns and potential research fatigue. Despite numerous measures taken by the research team to increase trust and response rate (training of data collectors, use of name badges, using a peer approach during snowball sampling etc.), 15 households refused to participate in the study and no information could be gathered about their demographic or other characteristics, which could have been different than the study population.

#### Referral for services

An additional challenge encountered was related to limited health care access for unregistered asylum seekers. Although respondents with significant psychological symptoms were routinely referred to available mental health services, the research team was able to do less for undocumented people, since they did not have free health care coverage and needed other means of support such as free psychosocial support delivered by NGOs.

#### Cultural barriers

The research team also experienced some cultural and gender-related challenges. For instance, women were reluctant to be interviewed by a male interviewer and it was not easy to find men at home during working hours, as most men were at work during daytime. To avoid bias, necessary measures were taken to end up with a gender-balanced sample (see below).

#### Resentment from host communities

The two neighborhoods where the study was conducted were among the most disadvantaged ones in Ankara and were also home to other (Afghan, Somali) refugees, as well as low-income Turkish families. There was visible animosity towards Syrian refugees by others, fueled by anti-immigrant propaganda (“they are taking up our jobs”, “they receive money from the government, etc.”). This factor did not prevent the research efforts, but may have diminished motivation of others in helping the interviewers find refugees and houses to include in the study.

#### Language

Language was another important challenge for this specific study topic and population, since mental health care generally requires more linguistic competence both on the patient and provider side. During the interviews, almost all respondents mentioned the language problem as the main reason for low contact with mental health services. A similar challenge existed for the researchers. First of all, none of the senior researchers spoke Arabic and most adult refugees did not speak Turkish. The different alphabet (Latin) used in Turkey added to the language barrier. Plus, most of the refugees in the present study had low educational attainment. These factors created several difficulties in conducting the study. First, the available options for choosing the study measures were limited. The research team had difficulty in finding appropriate depression and PTSD scales validated in Arabic language, mainly because no one in the team could follow the literature in Arabic. Second, bilingual field staff had to be employed in order to translate sociodemographic form (in Turkish) into Arabic. Differing dialects of Arabic posed another challenge in both translation and administration of the scales. Since most of the participants had low education, self-report questionnaire items needed to be read out and recorded, which led to longer interview durations. Finally, recruiting bilingual and educated interviewers with research skills was very difficult.

### Strategies used to address the challenges

#### Training of data collectors

Mental health research requires data collection on sensitive issues and data collectors need special communication skills or structured trainings to be able to collect good-quality data and to avoid any conflicts, bias or ethical problems during data collection in the field. Literature findings provide insights on the cascade of events that make professionals working with traumatized people especially vulnerable to secondary traumatic stress and broader psychological distress. Therefore, conducting trainings to develop skills specifically for mental health research is necessary to decrease the risk of secondary traumatization among early career researchers and data collectors [28, 29]. In the present study, strict criteria were set for recruitment of interviewers and standard training sessions were organized before data collection. In parallel to the previously set criteria by the research team, all interviewers had a health science background, had good communication skills, and they were residing in Turkey for at least four years prior to the study. The interviewers were recruited in a two-steps approach; i) an outreach email sent via the e-mail list server of the university, ii) face-to-face interviews with suitable applicants.

#### Sampling methodology

The initial approach was to use a probability sampling method to ensure a representative sample for the target group. However, discussions with local authorities showed that this was not feasible due to numerous external factors including lack of publicly available demographic data on a district level, the presence of unregistered asylum seekers and the high mobility among the registered refugee population. The research team therefore decided to work in two neighborhoods, known to be densely populated with refugees and use a snowball sampling technique to be able to reach out to the specific target group, avoiding unnecessary contact with other migrants and non-migrant households. Literature shows that finding appropriate sampling strategies to obtain representative results is challenging in migration research. Utilizing a snowball sampling method appears to be an effective strategy to outreach vulnerable or marginalized population groups. In addition, other methods such as cluster sample and respondent-driven sampling (RDS) are suggested in order to increase survey response in hard-to-reach populations [30–32].

#### Cultural considerations and gender-sensitive approach

Data collection procedure had some cultural and gender-related challenges. For example, women were reluctant to be interviewed by a male interviewer and it was not easy to find men at home during daytime and most days of the week. Therefore, the interviewer team was composed of both genders, and the interviews were conducted with a same-sex interview approach. Also, the field team had to work during several nights and weekends to be able to interview more men and have a gender-balanced sample at the end. Growing evidence from literature demonstrates that women and men differ significantly in susceptibility to health problems and response to treatment. Many refugee women are influenced by gender roles and expectations and they are exposed to gendered health systems and practices, particularly mental health and access to services. Therefore, it is of utmost importance that studies are conducted with a gender-sensitive approach [33–35].

#### Community participation and pilot testing

Within the mixed model approach, we interviewed several members of health authority and health care staff providing care to the refugees. Those interviews helped us understand the most pressing health care needs of the refugees. Before we started data collection, we interviewed refugees as part of a pilot study to better understand the context and to finalize the questionnaire. Finally, some of our interviewers themselves were refugees, who greatly contributed to our understanding of the population specific needs and increased the quality of our data collection and analysis. Literature shows that community participatory research approaches that include working with community leaders, cultural mediators, and civil society organizations, as well as using peer-to-peer methodologies in data collection, analysis, and reporting increase the quality of research studies with migrants and refugees [36–38].

#### Safety considerations

Lastly, several measures were taken to ensure safety of the respondents and the interviewers during household visits and night shifts. First, one of the senior researchers always accompanied and supervised the field team during data collection. Second, all interviewers had clearly visible identification badges with the university logo and carried ethical and institutional approval papers with them at all times. In addition, a WhatsApp group was formed among the field team for better coordination and increased security during data collection. By using digital technologies, the interviewers were able to e-communicate, geolocate themselves and update their location during the household visits.

Our experience might have several implications for the wider public health research and practice in other settings. First, collaboration between public health professionals and mental health professionals with clinical experience increases the quality of data collection and interpretation of findings in mental health research. Second, considering different and even innovative sampling approaches with a scientific basis as well as using community participatory approaches and peer-to-peer methodologies help in increasing participation of hard-to-reach populations in public health research and practice. Third, using digital technologies can facilitate data collection and communication during public health field work, in addition to geolocating team members and ensuring safety in settings affected by conflicts, disasters or other challenging circumstances.

## Strengths and limitations

Although Syrian refugees have been in Turkey since 2011 and the number of refugees has been increasing each year, most of the previous studies collected data from camp residents. The current study has overcome those limitations by selecting a community-based urban sample and assessing health services use, in addition to the rates of mental disorders.

Nevertheless, this study also had some limitations. First, we did not use a clinical interview, which would make it possible to reach definitive medical diagnoses. On the other hand, our interviewers administered the batch including self-administered scales as an interview, mainly because the education level of the sample was low. Another limitation of the study was that the HTQ version we used assessed DSM-IV (Diagnostic and Statistical Manual of Mental Disorders-IV) PTSD instead of DSM-5; this was because a valid and reliable instrument calibrated for DSM-5 was not available at the time of our study. The research team used a quantitative approach to collect data from refugees and a qualitative approach for service providers and decision makers. Although adding a qualitative approach could help with more in-depth data collection from refugees, collecting data in a visual or audio format was not allowed by authorities at the time of the study. Second, our main aim was to administer validated mental health scales in addition to assessing sociodemographic features and services use. The batch including the scales and the questionnaire was already too long and it could be very difficult to have additional qualitative interviews. We therefore decided to use a quantitative approach for refugees and a qualitative approach for service providers and decision makers.

Although we tried to obtain a representative sample, population sizes and distributions according to different neighborhoods were not available and our sample was not random. We had to use snowball sampling to reach respondents and therefore cannot generalize our findings to Syrian refugees in Turkey. We included all adults in the contacted households instead of choosing a random respondent, which may have created a bias in terms of prevalence of mental problems. On the other hand, each household usually had more than one family, sometimes going up to three or four families per household, which may have decreased the aforementioned bias. Despite training of data collectors by senior researchers for data collection to be standardized, there might still be interviewer bias encountered during actual field work.

The present study adds to the literature with its mixed-method approach when assessing services use, which made it possible to learn different points of views from refugees, health authorities and health service providers in a multidimensional manner. The research team disseminated the main findings to a variety of audiences including academia (via conference presentations and scientific publications), public sector, and national and international non-governmental organizations (NGOs and INGOs) (via presentations in health policy-oriented meetings). Our main aim in disseminating the findings was to inform future mental health policy-making and service provision in Turkey, in addition to advocate for increased efforts to promote refugee mental health by a multi-sectoral approach, including targeted interventions to improve social determinants of mental health [23–27].

## Conclusions

International literature shows that less is known about the mental health status of refugees living in urban settings when compared to camps. This might be partly due to the challenges of working with community-based refugee populations in host countries. The current study showed that research studies with refugees, especially research on mental health topics have their own methodological characteristics and challenges. The research team’s experience indicates that refugee population characteristics including level of trauma, language, culture, gender, legal status, and urban setting characteristics including places of living, mobility, availability of publicly available demographic data, and outreach-related barriers lead to different challenges and ethical responsibilities of researchers and affect the research costs in terms of time, human resources and finance. Even in a host country with geographical, religious and cultural proximity to the refugees, profound challenges exist in conducting mental health research in urban settings.

Some of the challenges we identified were similar to those found in other public health practices or research. International literature regarding humanitarian and migration research do show examples of difficulties encountered during sampling, recruiting, reaching out and working with refugee and migrant populations. Lack of registry data, language barriers, cultural and gender related differences, trust issues, and secondary traumatic stress have been discussed in other studies as well [26, 28, 30–38], however, there are also region and host country specific factors including geographical, legal, administrative and culturally sensitive issues that future researchers and practitioners need to take into account when planning to work in different humanitarian and forced migration settings.

Future studies with refugee populations in urban settings should take into account the contextual factors, as well as refugee and host population characteristics with a specific focus on language, culture, gender, living and working conditions. In order to overcome some of the barriers faced in the field, it is of utmost importance to use participatory research approaches and involve refugees in the research process beginning from the planning stage of the studies. Contacting local authorities and organizations working with/for refugees during planning stage can also help researchers understand the specific context and study population.

Migration researchers should aim to find innovative ways to implement community participatory research approaches such as working with community leaders and cultural mediators, in addition to using peer-to-peer methodologies in data collection, analysis, and reporting, as well as in doing advocacy for future policy making based on scientific evidence. Research capacity and skills building trainings for early career researchers and data collectors help prevent secondary traumatic stress and increase quality of collected data during research in forced migration settings. Lastly, collaboration with local researchers and institutions and acknowledging local knowledge and experience are vital for better public health research and practice outcomes.

## Data Availability

The datasets used and/or analyzed during the current study are available from the corresponding author on reasonable request.

## Abbreviations

UNHCR: United Nations High Commissioner for Refugees
PTSD: Post-Traumatic Stress Disorder
HTQ: Harvard Trauma Questionnaire
BDE: Beck Depression Inventory
DSM: Diagnostic and Statistical Manual of Mental Disorders
NGOs: Non-Governmental Organizations

## References

[1] UNHCR. Syria Emergency. https://www.unhcr.org/syria-emergency.html Accessed 03 June 2021.

[2] Alpak G, Unal A, Bulbul F, Sagaltici E, Bez Y, Altindag A, Dalkilic A, Savas HA (2015). Post-traumatic stress disorder among Syrian refugees in Turkey: a cross-sectional study. Int J Psychiatry Clin Pract.

[3] Porter M, Haslam N (2005). Predisplacement and postdisplacement factors associated with mental health of refugees and internally displaced persons: a meta-analysis. JAMA.

[4] Karadag O, Altintas KH (2010). Refugees and health. TAF Prev Med Bull.

[5] Bogic M, Njoku A, Priebe S (2015). Long-term mental health of war-refugees: a systematic literature review. BMC Int Health Hum Rights.

[6] Steel Z, Chey T, Silove D, Marnane C, Bryant RA, van Ommeren M (2009). Association of torture and other potentially traumatic events with mental health outcomes among populations exposed to mass conflict and displacement: a systematic review and meta-analysis. JAMA..

[7] Meeting Humanitarian Challenges in Urban Areas. Inter-Agency Standing Committee, 75th IASC Working Group Meeting, Initial Strategy Paper, 11–13 November 2009, UN-HABITAT Headquarters, Nairobi.

[8] Acarturk C, Cetinkaya M, Senay I, Gulen B, Aker T, Hinton D (2018). Prevalence and predictors of posttraumatic stress and depression symptoms among Syrian refugees in a refugee camp. J Nerv Ment Dis.

[9] Department General of Migration Management (2020) Migration statistics.https://engocgovtr/temporary-protection 27 Accessed 12 December 2020.

[10] Marwa K (2016). Psychosocial sequels of Syrian conflict. J Psychiatry.

[11] Chung MC, Alqarni N, Al Muhairi S, Mitchell B (2017). The relationship between trauma centrality, self-efficacy, posttraumatic stress and psychiatric co-morbidity among Syrian refugees: is gender a moderator?. J Psychiatr Res.

[12] Tekeli-Yesil S, Isik E, Unal Y, Aljomaa Almossa F, Konsuk Unlu H, Aker AT (2018). Determinants of mental disorders in Syrian refugees in Turkey versus internally displaced persons in Syria. Am J Public Health.

[13] Shoeb M, Weinstein H, Mollica R (2007). The Harvard trauma questionnaire: adapting a cross-cultural instrument for measuring torture, trauma and posttraumatic stress disorder in Iraqi refugees. Int J Soc Psychiatry..

[14] Kleijn WC, Hovens JE, Rodenburg JJ (2001). Posttraumatic stress symptoms in refugees: assessments with the Harvard trauma questionnaire and the Hopkins symptom Checklist-25 in different languages. Psychol Rep.

[15] Beck AT, Ward CH, Mendelson M, Mock J, Erbaugh J (1961). An inventory for measuring depression. Arch Gen Psychiatry.

[16] West J (1985). An Arabic validation of a depression inventory. Int J Soc Psychiatry.

[17] Abdel-Khalek AM (1998). Internal consistency of an Arabic adaptation of the Beck depression inventory in four Arab countries. Psychol Rep.

[18] Kilic C, Kaya E, Karadag Caman O, Uner S. Differential effects of war-related adverse and traumatic events on Syrian population in Turkey. International Society for Traumatic Stress Studies – poster abstract book p.12. ISTSS, 33rd annual meeting, 9-11 November 2017, Chicago, Illinois/USA.

[19] Kaya E, Karadag Caman O, Kilic C, Uner S. Need for and barriers to accessing mental health care among refugees in Turkey: a mixed methods study. Eur J Public Health. 2018;(Suppl.4):28. 10.1093/eurpub/cky213.453.

[20] Kaya E, Karadag Caman O, Kilic C, Uner S. Refugees' access to and utilization of health services: challenges and solutions in Turkey. Eur J Public Health. 2018;(Suppl.4):28. 10.1093/eurpub/cky214.273.

[21] Kaya E, Kilic C, Karadag Caman O, Uner S (2019). Posttraumatic stress and depression among Syrian refugees living in Turkey: findings from an urban sample. J Nerv Ment Dis.

[22] The UN Refugee agency-UNHCR. Figures at a Glance https://www.unhcr.org/afr/figures-at-a-glance.html. Accessed 02 August 2019.

[23] Craig T, Jajua P, Warfa N (2006). Mental healthcare needs of refugees. Psychiatry..

[24] Fazel M, Wheeler J, Danesh J (2005). Prevalence of serious mental disorder in 7000 refugees resettled in western countries: a systematic review. Lancet.

[25] de Anstiss H, Ziaian T, Procter N, Warland J, Baghurst P (2009). Help-seeking for mental health problems in young refugees: a review of the literature with implications for policy, practice, and research. Transcult Psychiatry.

[26] Cauce AM, Domenech-Rodriguez M, Paradise M, Cochran BN, Shea JM, Srebnik D (2002). Cultural and contextual influences in mental health help seeking: a focus on ethnic minority youth. J Consult Clin Psychol.

[27] Fenta H, Hyman I, Noh S (2006). Mental health service utilization by Ethiopian immigrants and refugees in Toronto. J Nerv Ment Dis.

[28] Živanović M, Vukčević MM (2020). Latent structure of secondary traumatic stress, its precursors, and effects on people working with refugees. PLoS One.

[29] Phillimore P, Sibai AM, Rizk A, Maziak W, Unal B, Abu Rmeileh N, Ben Romdhane H, Fouad FM, Khader Y, Bennett K, Zaman S, Mataria A, Ghandour R, Kılıç B, Ben Mansour N, Fadhil I, O’Flaherty M, Capewell S, Critchley JA (2019). Context-led capacity building in time of crisis: fostering non-communicable diseases (NCD) research skills in the Mediterranean Middle East and North Africa. Glob Health Action.

[30] Valerio MA, Rodriguez N, Winkler P, Lopez J, Dennison M, Liang Y, Turner BJ (2016). Comparing two sampling methods to engage hard-to-reach communities in research priority setting. BMC Med Res Methodol.

[31] Morris SK, Nguyen CK (2008). A review of the cluster survey sampling method in humanitarian emergencies. Public Health Nurs.

[32] Weinmann T, AlZahmi A, Schneck A, Mancera Charry JF, Fröschl G, Radon K (2019). Population-based assessment of health, healthcare utilisation, and specific needs of Syrian migrants in Germany: what is the best sampling method?. BMC Med Res Methodol.

[33] Sex and Gender Sensitive Research Call to Action Group, Wainer Z, Carcel C. Sex and gender in health research: updating policy to reflect evidence. Med J Aust. 2020;212(2):57–62.e1. doi: 10.5694/mja2.50426. Epub 2019 Nov 24. 10.5694/mja2.50426 PMC7027556 31760662

[34] Karadag Caman O and Bahar Ozvaris S. International Migration and Women’s Health. Saglik ve Toplum 2010; 20(4): 3-13. https://ssyv.org.tr/wp-content/uploads/2019/08/Uluslararas%C4%B1-Goc-ve-Kad%C4%B1n-Sagl%C4%B1g%C4%B1.pdf accessed 14 ember 2020.

[35] Zivot C, Dewey C, Heasley C, Srinivasan S, Little M (2020). Exploring the state of gender-centered Health Research in the context of refugee resettlement in Canada: a scoping review. Int J Environ Res Public Health.

[36] Hernando C, Sabidó M, Casabona J (2018). Facilitators and barriers of participation in a longitudinal research on migrant families in Badalona (Spain): a qualitative approach. Health Soc Care Community.

[37] Jewett-Tennant J, Collins C, Matloub J, Patrick A, Chupp M, Werner JJ, Borawski EA (2016). Partnership among peers: lessons learned from the development of a community organization-academic research training program. Prog Community Health Partnersh.

[38] Enticott JC, Shawyer F, Vasi S, Buck K, Cheng IH, Russell G, Kakuma R, Minas H, Meadows G (2017). A systematic review of studies with a representative sample of refugees and asylum seekers living in the community for participation in mental health research. BMC Med Res Methodol.

